# Mini Review of Controlled Cortical Impact: A Well-Suited Device for Concussion Research

**DOI:** 10.3390/brainsci7070088

**Published:** 2017-07-20

**Authors:** Nicole Osier, C. Edward Dixon

**Affiliations:** 1 School of Nursing, Holistic Adult Health Division, University of Texas at Austin, Austin, TX 78701, USA; nicoleosier@utexas.edu; 2 Dell Medical School, Department of Neurology, University of Texas at Austin, Austin, TX 78701, USA; 3 Safar Center for Resuscitation Research, University of Pittsburgh, Pittsburgh, PA 15224, USA; 4 Department of Neurological Surgery, University of Pittsburgh, Pittsburgh, PA 15260, USA; 5 VA Pittsburgh Healthcare System, Pittsburgh, PA 15240, USA

**Keywords:** controlled cortical impact (CCI), traumatic brain injury (TBI), experimental TBI, concussion, mild TBI (mTBI)

## Abstract

Mild traumatic brain injury (mTBI) is increasingly recognized as a significant public health problem which warrants additional research. Part of the effort to understand mTBI and concussion includes modeling in animals. Controlled cortical impact (CCI) is a commonly employed and well-characterized model of experimental TBI that has been utilized for three decades. Today, several commercially available pneumatic- and electromagnetic-CCI devices exist as do a variety of standard and custom injury induction tips. One of CCI’s strengths is that it can be scaled to a number of common laboratory animals. Similarly, the CCI model can be used to produce graded TBI ranging from mild to severe. At the mild end of the injury spectrum, CCI has been applied in many ways, including to study open and closed head mTBI, repeated injuries, and the long-term deficits associated with mTBI and concussion. The purpose of this mini-review is to introduce the CCI model, discuss ways the model can be applied to study mTBI and concussion, and compare CCI to alternative pre-clinical TBI models.

## 1. Introduction

Mild traumatic brain injury (mTBI) is a type of neurological event that is gaining international recognition as a significant worldwide public health problem which warrants further research. Some mTBIs are accompanied by concussion, a neurological syndrome with characteristic transient cognitive, motor, and neuropsychiatric symptoms [1]. While the symptoms associated with concussion typically resolve in the minutes-to-hours after injury [2,3,4], post-concussive syndrome, a neurological sequela [1], can occur and is associated with longer-term deficits. Still, despite the prevalence and increased interest in both mTBI and concussion, important gaps in the knowledge base remain, surrounding: characterizing the pathophysiology, understanding the factors that contribute to functional deficits, and developing targeted interventions capable of reducing pathology and symptoms. Pre-clinical models of TBI supplement accumulating clinical knowledge and remain an important part of the effort to generate evidence about both mTBI and concussion. There are several pre-clinical models that have been applied to study mTBI and concussion; each has unique strengths and limitations. The purpose of this mini-review is to provide an overview of the controlled cortical impact (CCI) model, discuss features that make it well-suited to studying mTBI and concussion, as well as compare CCI to available alternatives.

## 2. Model Overview

Animal models have been employed to study mTBI and concussion since the late 1800s, with efforts to refine and expand the methods in the subsequent years [5,6,7,8,9,10,11]. The first CCI paper published in 1988 used ferrets as the test animal [12]. During the 1990s, the model’s strengths (e.g., control and reproducibility) led others to adapt the model to rats [13], and later mice [14]. In the decades since, CCI has become a widely used model of experimental TBI that has been adapted for use in larger laboratory animals [15,16,17], as described in detail later (see Section 2.2). In addition to the expansion to different test animals, CCI has been modified to model closed head injury and repeated injuries (discussed in Section 3.4).

### 2.1. CCI Devices

CCI devices induce injury by using a pneumatic piston or electromagnetic actuator to drive an injury induction tip of the desired size/shape, with a specified velocity, depth, and dwell time into the exposed dura or intact skull. The use of a stereotaxic frame allows researchers to choose whether the tip is perpendicular or angled with respect to the injury site. The CCI model was initially characterized and refined using pneumatic devices [12,13,14]. Today, pneumatic CCI devices remain popular [18,19,20,21,22], with several commercially available models. Notably, electromagnetic devices have become commercially available and have been adopted by many research teams [23,24,25,26]. Both pneumatic and electromagnetic CCI devices have been well-received by the research community. Notably, there is little empirical evidence formally comparing pneumatic and electromagnetic devices. One published study did test one commercially available pneumatic and one prototype electromagnetic device and found greater reproducibility with the electromagnetic device [23]. Specifically, the authors found the pneumatic device resulted in velocity-dependent overshoot (which was not seen in the electromagnetic model) and greater overall overshoot [23]. Notably, the proper use of either a pneumatic or electromagnetic CCI device can produce graded [23,24,27,28,29], reproducible injuries that model important features of mTBI and concussion that are seen in humans.

### 2.2. Test Animals

CCI can be adapted to scale for smaller and larger animals, contributing to the model’s popularity. The initial ferret model [12] was scaled down for use in rats [13], and then further scaled down to account for the thinner cortex of mice [14]; the use of ferrets declined and smaller rodent models became the norm. Later, CCI was scaled up for use in larger laboratory animals, including nonhuman primates [17] and swine [15,16,30,31,32,33]. Ferret models have recently regained popularity with two new papers published recently [34,35].

Generally speaking, while controlling for the desired injury severity, the tip size and impact depth are scaled up with the size of the test animal. When conducting CCI in a test animal not used previously, researchers are advised to complete a thorough review of the literature and conduct pilot studies to characterize the model for the desired injury extent and specific outcomes of interest.

Moreover, the experimental endpoints of interest influence the selection of which test animal to use. As part of the effort to minimize animal suffering through reduction, replacement, and refinement, the least sentient animal appropriate to address the experimental goals should be used. Still, there are times when the use of animals with larger brain mass is needed, such as to test the effects of focal brain cooling devices or extracranial brain stimulation. When larger animals are used, additional approvals, regulations, and restrictions should be followed, as appropriate. Other considerations regarding choosing a test animal include housing, husbandry, behavioral testing options, and cost. 

## 3. Applications for Studying Mild TBI and Concussion with CCI

### 3.1. Overview of CCI for Studying mTBI and Concussion

Just as CCI can be scaled down to accommodate a smaller test animal, the injury can also be scaled within a given test animal to achieve a more mild injury. The remainder of this mini review will be devoted discussing applications of CCI for studying mTBI and concussion. As a disclaimer, there is no consensus regarding how to precisely set the CCI injury parameters to induce mTBI or concussion and different laboratories use different operationalizations. It is important to note that the terms mTBI and concussion are sometimes erroneously used interchangeably, though there are important distinctions, with mTBI being a neurological event and concussion being a neurological syndrome [1]; the use of the terms mTBI or concussion in this mini review will be based on the terms used in the primary source.

### 3.2. Open Head Mild TBI

Traditionally, CCI follows anesthetized craniectomy and the impactor tip contacts the exposed dura to induce injury. This traditional approach has been used in several studies to model mild TBI or concussion [18,36,37,38]. Outcomes of interest in these open head CCI studies is diverse, and has evolved over time to range from cortical perfusion responses [39] to gene expression changes [40]; behavioral deficits have also been explored in this context [22,38].

Open head CCI has also been used in combination with knockout (KO) animals to study the effects of gene and gene products after mild TBI or concussion. One study induced mild CCI in adenosine A(1) receptor (A(1)AR) KO mice (vs. wildtypes) and found that A(1)AR KO was associated with a greater microglial response after mTBI, as evidenced by a 20–50% increase in Iba-1+ microglia in KO animals [37]. Another study used an electromagnetic CCI device to induce mild TBI in mice deficient in cyclooxygenase (COX)-1 or COX-2 (vs. wildtypes of the same background strain) and compare histopathological and cognitive outcomes based on genotype; interestingly, cognitive deficits were not associated with knockouts of either gene [38].

### 3.3. Closed Head Mild TBI

Many TBIs, especially mTBI, do not result in damage to the skull or breaching of the dura. Thus, there has been an increased effort to create pre-clinical models of closed head injury (CHI) to better study mild TBIs and concussions. Part of this effort includes adaptations of the CCI model to eliminate the craniectomy and deliver the impact to the closed skull [41,42,43,44]. One study tested the effects of mild CCI over the left lateral skull and the effects of sphingosine 1-phosphate (S1P) or rolipram treatment, and found beneficial effects to immune suppression soon after mTBI [45]. Another study using the same pneumatic model found beneficial effects of low-level light therapy on inflammatory outcomes after CHI [46]. In a follow-up, the team identified that the therapeutic effect of low-level light after mTBI can be enhanced by adding metabolic modulators [21].

### 3.4. Repeated mTBIs or Concussions

As the consequences of multiple head traumas have become of increasing concern, CCI has also been extended to study multiple injuries. This line of inquiry is relevant to those at risk for repeated mTBIs and/or concussions, such as people involved in certain sports or military roles. Part of the effort to understand the consequences of repeated traumas has been studied, comparing the consequences of single vs. multiple CCI [42,44]. In one recent closed head CCI study [42], features of the CCI model were combined with those from Marmarou’s impact acceleration model and single or multiple TBIs delivered; performance on several behavioral tests was more impaired with repeated injury. In a rat model of single vs. repeated concussion induced using a closed skull TBI model, acute outcomes were similar regardless of the number of concussions; however, the duration of memory deficits was longer in rats exposed to repeated concussions [44]. 

## 4. Alternative Models and Limitations

In addition to CCI, there are a number of other experimental TBI models that can be used to study various TBI severities, including mTBI and concussion. Popular alternatives are fluid percussion injury (FPI) and weight drop injury (WDI), though numerous additional alternatives exist. Notably, there are very few studies that empirically compare models of TBI, and of those that have been published [47,48,49,50], the majority compare models of moderate-to-severe TBI. One study compared mild CCI to mild FPI and found that there was substantial overlap in the genes dysregulated after both forms of mild injury [40]. However, mild FPI resulted in additional dysregulation beyond what was seen in mild CCI [40]; this may be due to the diffuse nature of the FPI model compared to the focal CCI model. Each model has its own sets of strengths and limitations which need to be considered when selecting the appropriate model for a given research application or outcome(s) of interest.

There are limitations common to all experimental TBI models. While animals do model important aspects of TBI pathophysiology and functional deficits, they cannot completely replicate the pathophysiology and symptoms profiles experienced by humans with TBI. As described in a recent review, the commonly used experimental TBI models each have a set of experimental endpoints they model well, while other endpoints are either not as effectively modeled or remain to be empirically validated [51]. Also, most pre-clinical TBI models use anesthesia to reduce animal suffering, which has potentially confounding effects, although the use of sham (vs. naïve) controls helps to temper this concern.

Limitations specific to CCI also exist. For example, CCI produces a focal contusion-type injury. While some mild TBIs have been found to result in contusive injuries such as TBIs caused by motorcycle accidents [52] and those occurring in mixed martial arts (MMA) fighters [53], other mild TBIs tend to produce more widespread concussive injuries accompanied by the loss of consciousness [53]. Whereas the variety of options regarding tip geometry (e.g., size, shape) and injury parameters (e.g., depth, dwell time, velocity) add to the customizability of the CCI model, these options also make comparisons across studies challenging. This underscores the importance of describing relevant experimental details as outlined in the National Institute of Neurological Diseases and Stroke’s Common Data Elements (CDEs), which highlight reportable information common to all pre-clinical TBI studies as well as that which is specific to CCI studies [54].

## 5. Conclusions

In its three decades of use, CCI has become a popular experimental TBI model used in several species of test animal for numerous research applications. The scalability of the CCI model can be harnessed to study the consequences of and treatments for both single as well as repeated mTBI(s) or concussion(s). While there are numerous ways to model mTBI and concussion in animal models, CCI remains a popular choice.
